# Failing the four-gamete test enables exact phasing: the Corners’ Algorithm

**DOI:** 10.1186/s12711-022-00763-1

**Published:** 2022-11-14

**Authors:** Luis Gomez-Raya, Wendy M. Rauw

**Affiliations:** Departamento de Mejora Genética Animal, Instituto Nacional de Investigación y Tecnología Agraria y Alimentaria (INIA-CSIC), Ctra de La Coruña Km 7.5, 28040 Madrid, Spain

## Abstract

**Background:**

Failing the four-gamete test for two polymorphic DNA markers is an indication that two or three rather than four haplotypes segregate in the population. The objective of this paper is to show that when just three haplotypes are segregating, all three haplotypes can be fully and unambiguously phase-resolved.

**Theory and methods:**

The Corners’ Algorithm tests the four corners in a 3 × 3 table of two-locus genotypes. If one of the four corners is filled with zeroes, then the missing haplotype is identified and the phases of all three haplotypes can be unambiguously resolved for all individuals. Three applications of this method are proposed when the four-gamete test fails: (1) direct estimation of linkage disequilibrium (LD), (2) haplotype-based genome-wide association studies (GWAS) of three haplotypes (single-marker GWAS tests for two out of three haplotypes only), and (3) haplotyping of chromosomal regions that are comprised of pairs of single nucleotide polymorphisms (SNPs) that consist of just three haplotypes. An example based on 435 sows with performance records for total number of piglets born is used to illustrate the methods.

**Results:**

Of 20,339 SNPs, approximately 50% of the pairs of flanking SNPs failed the four-gamete test. For those, the expectation maximization (EM) algorithm gave the same results. The average of the absolute value of the difference in *r*^*2*^ between flanking SNPs across the genome between the two methods was 0.00082. Single-marker GWAS (using two of three haplotypes) detected significant associations for total number of piglets born on chromosomes 1, 2, 6, 9, 10, 12, 13, 14, 15, and 18. Haplotype-based GWAS using the third haplotype resolved with the Corners’ Algorithm detected additional significant associations for total number of piglets born on chromosomes 2, 5, 10, 13, 14, 15, and 18. Estimated substitution effects ranged from 0.40 to 1.35 piglets. Haplotyping of chromosomal regions that failed the four-gamete test for any pair of SNPs covered 961 Mb out of the 2249 Mb by the SNP array.

**Conclusions:**

The Corner’s Algorithm allows to fully phase haplotypes when the four-gamete test fails. Longer haplotypes in chromosomal regions in which the four-gamete test fails for any pair of SNPs can be used as a multi-allelic marker with increased polymorphism information content.

**Supplementary Information:**

The online version contains supplementary material available at 10.1186/s12711-022-00763-1.

## Background

Professor Rohan Fernando, together with Professor Grossman, made one of the earliest theoretical contributions to incorporate marker information to traditional pedigree information [1]. They provided equations to compute the covariance between relatives conditional on pedigree and single-marker information. Their pioneering work was performed in 1989 when availability of markers was rather poor and when no one could envision how the landscape of research in genetics would drastically change in just three decades with emerging technologies, such as DNA sequencing and genotyping by arrays of densely positioned single nucleotide polymorphisms (SNPs) that are in gametic phase linkage disequilibrium (LD) with each other and likely with causal loci. Linkage disequilibrium is the non-random association of alleles at two or more loci [2]. It has a great impact on the application of all molecular technologies that attempt to relate genotypes with disease or performance traits across species, including farm animals and humans.

Let us consider two polymorphic loci, *A/a* and *B/b*, which result in four possible haplotypes, *AB*, *Ab*, *aB,* and *ab*. Linkage disequilibrium is commonly defined as $D={f}_{AB}{f}_{ab}-{f}_{Ab}{f}_{aB}= {f}_{AB}{-f}_{A}{f}_{B}$, where ${f}_{i}$ is the frequency of the $i$*-th* haplotype ($i$ = *AB*, *Ab*, *aB*, *ab*). Consequently, the allele frequencies of *A* and *B* at the two loci are ${f}_{A}={f}_{AB}+{f}_{Ab}$, and ${f}_{B}={f}_{AB}+{f}_{aB}$, respectively. Estimation of LD in diploid species requires resolving haplotype phases for the individuals in the population by direct or inferential methods. Direct methods use specialized experimental techniques applied to genomic DNA derived from a single individual [3, 4], while inferential methods use statistical means to infer haplotypes. In populations of unrelated individuals from a diploid species, LD is often determined using the expectation maximization (EM) algorithm, which assumes Hardy–Weinberg equilibrium [5]. This algorithm is iterative and requires initial estimates of haplotype frequencies to converge to the maximum likelihood estimates. Alternatively, empirical correlation between allele dosages (0, 1, 2) can also be used to estimate LD but it does not provide phasing information. The main problem in resolving haplotypes in a two-locus system in diploids is that the haplotype phase in double heterozygous individuals can only be determined with an associated probability, that is, phases are not exact. Bayesian methods implemented in the software PHASE can improve accuracy of phasing of haplotypes but still does not unambiguously resolve all individual haplotypes [6, 7]. A review of existing phasing methods is given by Browning and Browning [8].

Given the allele frequencies, the maximum absolute value that LD (*D*) for a pair of loci can attain occurs in five cases:

(I) Allele *A* is fully associated with allele *b* ${(f}_{A}={f}_{Ab}$), while allele *a* is partially associated with both *B* and *b* alleles, and therefore, only three haplotypes (*Ab*, *aB*, *ab*) are segregating in the sample;

(II) Allele *A* is fully associated with allele *B* ${(f}_{A}={f}_{AB}$), while allele *a* is partially associated with both *B* and *b* alleles, and therefore, only three haplotypes (*AB*, *aB, ab*) are segregating in the population;

(III) Allele *a* is fully associated with allele *b* ${(f}_{a}={f}_{ab}$), while allele *A* is partially associated with both *B* and *b* alleles, and therefore, only three haplotypes (*ab*, *AB*, *Ab*) are segregating;

(IV) Allele *a* is fully associated with allele *B* ${(f}_{a}={f}_{aB}$), while allele *A* is partially associated with both *B* and *b* alleles, and therefore, only three haplotypes (*aB*, *AB*, *Ab*) are segregating;

(V) Allele *A* is fully associated with allele *B* ${(f}_{A}={f}_{B}={f}_{AB}$), while allele *a* is fully associated with allele *b* ${(f}_{a}={f}_{b}={f}_{ab}$), and therefore, only two haplotypes (*AB* and *ab*) are segregating; The same argument can be made for the full LD of allele *A* with *b* (haplotypes *Ab* and *aB*).

These five cases result in maximum LD given the allele frequencies because in the formula for calculating $D$, the product of either ${f}_{AB}{f}_{ab}$ or ${f}_{Ab}{f}_{aB}$ is zero in each of these five cases. Consequently, given the allele frequencies, segregation of only two or three haplotypes results in maximum LD. This situation fails the four-gamete test, which detects recombination events with four segregating haplotypes that cannot have arisen without either recombination or a repeat mutation. Hudson and Kaplan [9] defined the four-gamete test as “For the infinite site model, the mutation rate for any site is infinitesimal; therefore, at most one mutation event can occur in the history of the sample at that site. Thus, for any two sites there are at most four gametic types in the population. Furthermore, since the model does not allow for back mutation and recurrent mutation, the only way for all four gametic types to be present in the sample is for at least one recombination event to have occurred in the history of the sample between the two sites”. In practical terms, failing the four-gamete test means that there are less than four haplotypes segregating in a two-locus system.

Lewontin [10] proposed another measure of LD, $D^{\prime}$, as the ratio of $D$ to its maximum possible absolute value, given the allele frequencies. If $D^{\prime}=1$, at least one of the four possible haplotypes must be absent, regardless of allele frequencies. Today, the most widely used method to measure LD is ${r}^{2}$ [11], which is defined as the square of the correlation between locus allele dosages in the segregating haplotypes:

1 $$r^{2}=\frac{D^{2}}{f_{A} f_{a} f_{B} f_{b}}.$$

This measure is widely used in spite of its dependence on allele frequencies [12, 13]. The values of ${r}^{2}$ range from 0 to 1. The latter value can only occur when either ${f}_{A}={f}_{B}$, or ${f}_{A}={1-f}_{B}$. In fact, in many instances and depending on allele frequencies, the maximum ${r}^{2}$ may take values much lower than 1 [14].

In traditional single-marker genome-wide association studies (GWAS), a measure of the association or statistical dependence between the number of copies for one of the alleles at a SNP and the phenotype investigated is computed and repeated for each SNP. A large number of SNPs sparsely distributed across the genome are used to identify genotype–phenotype associations [15]. GWAS has triggered a vast number of studies that aimed at identifying genes that are responsible for diseases in humans [16]. In spite of its success, GWAS often explains only a small fraction of the observed phenotypic variability, a phenomenon referred to as the missing heritability [17, 18]. Most GWAS have considered one marker at a time but there is great interest and much research efforts in considering multiple makers and/or haplotypes in GWAS [19]. Exact phasing benefits the use of multi-marker GWAS by assigning haplotypes unambiguously to each individual.

The main objective of this study is to show that failing the four-gamete test (i.e., presence of less than four haplotypes) enables exact phasing by a newly developed Corners’ Algorithm. It enables the identification of haplotypes in two-locus systems. Our other objectives are to show applications of exact phasing for situations where the four-gamete test fails: (1) direct estimation of LD, (2) GWAS using haplotypes, and (3) haplotyping of chromosomal regions. An example using Iberian sows that are typed with a low-density SNP array and for which total number born records are available is used to illustrate the proposed methods.

## Methods

### Theory

#### Exact phasing of haplotypes when the four-gamete test fails: the Corners’ Algorithm

Genotype counts at two loci (${n}_{ij}$, $i$ = *AA*, *Aa*, *aa*; $j$ = *BB*, *Bb*, *bb*) can be arranged in a 3 × 3 table as shown in Table 1. If one of the four haplotypes is not existing, there will be no observations in one of the four corners. Which of the four corner holds zeroes depends on which of the four haplotypes is missing: haplotypes *AB*, *Ab*, *aB*, and *ab* for corners I, II, III, and IV, respectively (Table 2). Note that for all genotype pairs, with the exception of ${n}_{AaBb}$ in the center of the table, the haplotype phase can be unambiguously resolved. It must be assumed that corners with zeroes occur because their corresponding double genotypes (and corresponding haplotypes) are missing in the population. It is possible that those double genotypes are just not observed in our sample. However, if the sample size is not small then the chance that double genotypes corresponding to a corner would be observed in the sample but existing in the population is extremely low.

**Table 1:** Notation for genotype counts at two loci with alleles *A/a* and *B/b,* ${{n}}_{{i}{j}}$ (${i}$ = *AA, Aa, aa;* ${j}$ = *BB, Bb, bb*)

	*BB*	*Bb*	*bb*
*AA*	*n*_*AABB*_	*n*_*AABb*_	*n*_*AAbb*_
*Aa*	*n*_*AaBB*_	*n*_*AaBb*_	*n*_*Aabb*_
*aa*	*n*_*aaBB*_	*n*_*aaBb*_	*n*_*aabb*_

**Table 2:** Possible two-locus genotype counts when one of the four possible haplotypes (*AB*, *Ab*, *aB*, *ab*) is missing, as determined by a zero genotype count at one of the four corners of the genotype count table

Corner I, Missing haplotype *AB*, double heterozygotes are phased *Ab/aB*			
	*BB*	*Bb*	*bb*
*AA*	*0*	*0*	*n*_*AAbb*_
*Aa*	*0*	*n*_*AaBb*_	*n*_*Aabb*_
*aa*	*n*_*aaBB*_	*n*_*aaBb*_	*n*_*aabb*_

Corner II, Missing haplotype *Ab*, double heterozygotes are phased *AB/ab*			
	*BB*	*Bb*	*bb*
*AA*	*n*_*AABB*_	*0*	*0*
*Aa*	*n*_*AaBB*_	*n*_*AaBb*_	*0*
*aa*	*n*_*aaBB*_	*n*_*aaBb*_	*n*_*aabb*_

Corner III, Missing haplotype *aB*, double heterozygotes are phased *AB/ab*			
	*BB*	*Bb*	*bb*
*AA*	*n*_*AABB*_	*n*_*AABb*_	*n*_*AAbb*_
*Aa*	*0*	*n*_*AaBb*_	*n*_*Aabb*_
*aa*	*0*	*0*	*n*_*aabb*_

Corner IV, Missing haplotype *ab*, double heterozygotes are phased *Ab/aB*			
	*BB*	*Bb*	*bb*
*AA*	*n*_*AABB*_	*n*_*AABb*_	*n*_*AAbb*_
*Aa*	*n*_*AaBB*_	*n*_*AaBb*_	*0*
*aa*	*n*_*aaBB*_	*0*	*0*

When at least one of the corners has zero observations, double heterozygotes, *AaBb*, with genotype count ${n}_{AaBb}$, can be unambiguously phased by the Corners’ Algorithm that comprises the following steps:

1. Set the genotype counts for two loci in a 3 × 3 table as shown in Table 1.
2. Identify any of the four possible situations with one corner without observations, as portrayed in Table 2 (corner I, II, III, or IV). If two opposite corners in the table have zero observations (either I and III, or II and IV), then two haplotypes are missing (only two haplotypes are segregating) and the two loci are in complete LD.
3. Identify the missing haplotype corresponding to missing observations in corner I, II, III, or IV.
4. Resolve the linkage phase unambiguously for all individuals, including double heterozygotes. This can be done since the haplotypes in Table 2 are only possible when that given non-observed haplotype is excluded (haplotype *AB*, *Ab*, *aB*, and *ab* for corner I, II, III, and IV, respectively). Specifically, double heterozygotes can be phased as either *AB/ab* or *Ab/aB*. However, if haplotype *AB* is not observed in corner I, since none of *AABB*, *AABb* or *AaBB* are observed, all double heterozygotes must be phased as *Ab/aB.* The same argument can be made for the other corners.

#### Direct estimation of LD when the four-gamete test fails

Phasing all haplotypes when the four-gamete fails allows direct estimation of ${r}^{2}$, as described in the following. Consider corner I in Table 2, where the non-observed haplotype is *AB*. Computing haplotype frequencies from two-locus genotype counts is straightforward when recognizing that the double heterozygote must be phased as *Ab/aB*:

$${f}_{AB}=0,$$

$${f}_{Ab}=\frac{2{n}_{AAbb}+{n}_{AABb}+{n}_{Aabb}+{n}_{AaBb}}{2N},$$

$${f}_{aB}=\frac{2{n}_{aaBB}+{n}_{AaBB}+{n}_{aaBb}+{n}_{AaBb}}{2N},$$

$${f}_{ab}=\frac{2{n}_{aabb}+{n}_{aaBb}+ {n}_{Aabb}}{2N}.$$

Resulting allele frequencies are:

$${f}_{A}=\frac{2{n}_{AA}+{n}_{Aa}}{2N},$$

$${f}_{a}=\frac{2{n}_{aa}+{n}_{Aa}}{2N},$$

$${f}_{B}=\frac{2{n}_{BB}+{n}_{Bb}}{2N},$$

$${f}_{b}=\frac{2{n}_{bb}+{n}_{Bb}}{2N},$$

where genotypic counts for the three genotypes for each locus are:

$${{n}_{AA}=n}_{AABB}+{n}_{AABb}+ {n}_{AAbb};$$

$${{n}_{Aa}=n}_{AaBB}+{n}_{AaBb}+ {n}_{Aabb};$$

$${n}_{aa}={n}_{aaBB}+{n}_{aaBb}+ {n}_{aabb};$$

$${{n}_{BB}=n}_{AABB}+{n}_{AaBB}+ {n}_{aaBB};$$

$${n}_{Bb}={n}_{AABb}+{n}_{AaBb}+ {n}_{aaBb};$$

$${n}_{bb}={n}_{AAbb}+{n}_{Aabb}+ {n}_{aabb};$$

and the total number of individuals is: *N* = ${n}_{AA}+{n}_{Aa}+{n}_{aa}={n}_{BB}+{n}_{Bb}+{n}_{bb}$.

Substituting these values into Eq. (1) yields:

$${r}^{2}={\left[{-\frac{(2{n}_{AAbb}+{n}_{AABb}+{n}_{Aabb}+{n}_{AaBb})(2{n}_{aaBB}+{n}_{AaBB}+{n}_{aaBb}+{n}_{AaBb})}{\sqrt{(2{n}_{AA}+{n}_{Aa})(2{n}_{aa}+{n}_{Aa})(2{n}_{BB}+{n}_{Bb})(2{n}_{bb}+{n}_{Bb})}}}\right]}^{2}.$$

LD is computed in the same way for the three other situations, with missing haplotypes *Ab*, *aB*, or *ab* in corners II, III, and IV (see Appendix).

#### Estimation of haplotype-based GWAS when the four-gamete test fails

**Fig. 1:**
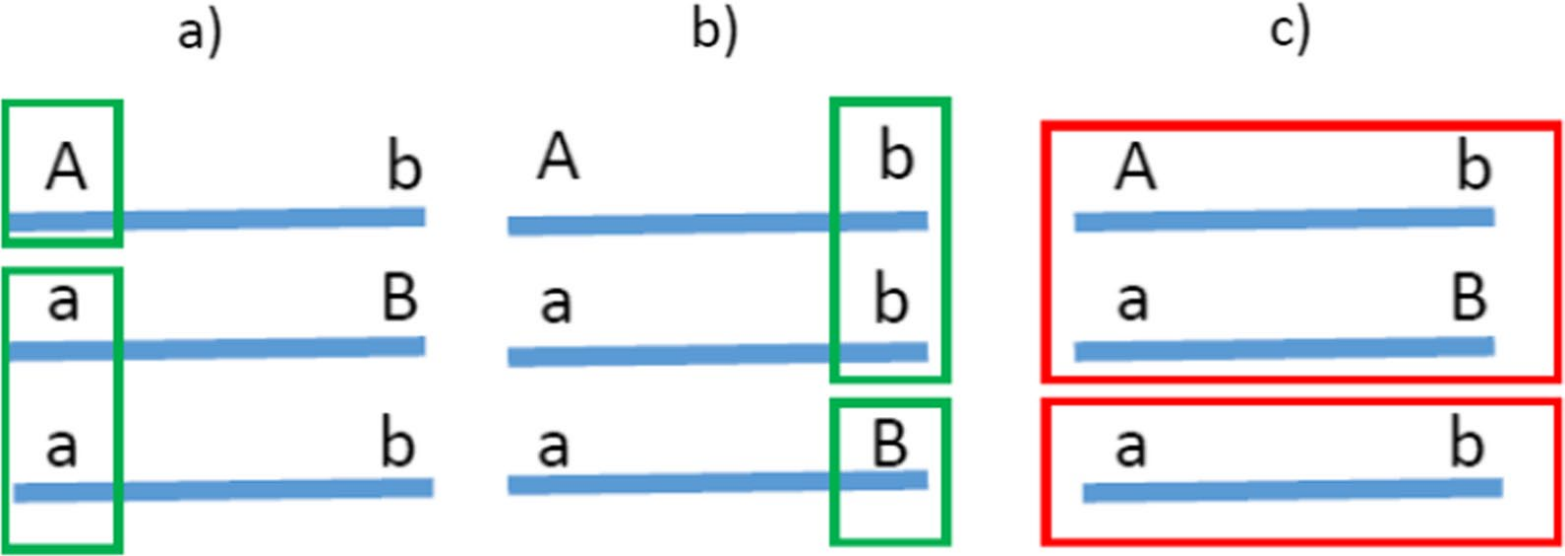
Contrast between haplotypes in single-marker GWAS *vs*. haplotype GWAS when all three haplotypes are resolved with the Corners’ Algorithm. **a** and **b** represent the contrast between the first marker and the second marker (contrast of haplotypes and alleles in green), respectively; **c** represents the contrast in the third haplotype that can be phase-resolved by the Corners’ Algorithm (in red)

The three unambiguously and fully phased two-locus haplotypes for all individuals facilitates the application of haplotype-based GWAS instead of single-marker GWAS to test for association with performance or disease. Consider the single-marker GWAS that tests for the association between a single SNP genotype with performance or disease (Fig. 1). By testing SNP *A*/*a*, given a pair of consecutive SNPs (*A*/*a* and *B*/*b*) and the missing haplotype *AB*, the single-SNP GWAS compares the performance of all individuals with haplotype *Ab* (i.e., with allele *A*) with that of all individuals with haplotypes *aB* and *ab* (i.e., with allele *a*: Fig. 1a). Likewise, when SNP *B*/*b* is tested, the performance of individuals with haplotype *aB* (i.e., with allele *B*) is compared to individuals with haplotypes *Ab* and *ab* (i.e., with allele *b*: Fig. 1b). This means that haplotypes *Ab* and *aB*, but not haplotype *ab* are contrasted against the two other haplotypes. Thus, the single-SNP GWAS method does not take into account that haplotype *ab* may have a different effect on phenotype than haplotypes *Ab* and *aB*. We propose to use haplotypes that are unambiguously phased with the Corners’ Algorithm, to test the performance of the third haplotype, *ab* (Fig. 1c). This is achieved by attributing 0, 1, or 2 copies of the third haplotype to each individual, and by comparing the performance of individuals accordingly to the number of copies of this haplotype that they carry. This test assumes that each haplotype is genetically identical among individuals, i.e., individuals that share the same haplotype have the same DNA sequence within the fragment.

#### Haplotyping of chromosomal regions that fail the four-gamete test for any combination of SNP pairs

When haplotypes for any pair of SNPs in a chromosomal segment can be resolved for all individuals with the Corners’ Algorithm, the phasing can be extended to the rest of the chromosomal fragment. The strategy for phasing is:

1. Apply the Corners’ Algorithm to each pair of consecutive and nonconsecutive SNPs within a chromosomal fragment. When the four-gamete test fails for any pair of consecutive or nonconsecutive SNPs, the absence of recombination along the entire fragment is assured in the individuals of the sample;
2. Confirm that all pairs of SNPs have either two or three (but not four) haplotypes;
3. Phase the heterozygous haplotypes for each pair of SNPs for each individual using the Corners’ Algorithm;
4. Align all haplotype phases for all heterozygous SNPs for the entire chromosomal fragment for each individual using the information from point (3);
5. Haplotypes for each individual are then completed by filling the alleles for which each individual is homozygous for any SNP within the fragment.

### Animal material

Genotypes and phenotypes of total number of piglets born for 435 sows from a composite line of Iberian pigs, Torbiscal, were used to illustrate our methods. The Torbiscal line resulted from the blending of four ancient Spanish and Portuguese Iberian strains and was genetically isolated between 1963 and 2013 [20].

### SNP genotyping

DNA was isolated from blood using a standard phenol/chloroform protocol. Samples were genotyped with the Illumina Porcine SNP60 BeadChip [21] and the Infinium HD Assay Ultra protocol (Illumina Inc.). Genotypes at 62,163 SNPs were analyzed with the Genome Studio software (Illumina) using the Sscrofa10.2 assembly, which was the reference map available at the time of genotyping. Data quality control was performed according to the following criteria: the call rate of the sample had to be higher than 0.96; and SNPs were retained if they had a call rate higher than 0.99; a GenTrain score higher than 0.70; and an ABR mean higher than 0.35. SNPs located on the sex chromosomes or with at least one inconsistent inheritance from dam to daughter were also removed. This filtering resulted in 26,359 remaining SNPs. The next step was to move from assembly Sscrofa10.2 to Sscrofa11.1 based on the SNP name. In total, 1054 SNPs were removed because they were not present on both reference maps. In addition, 2447 SNPs had missing genotypes for one or more sows and were removed for all animals. This was necessary so that all animals could be used to construct haplotypes in chromosomal segments that failed the four-gamete test, as missing information in one SNP for one individual prevents exact phasing of the haplotypes for that individual. In total, 2519 SNPs with a MAF lower than 0.05 were removed, leaving 20,339 polymorphic SNPs for further analyses. Therefore, all analyses were performed with SNP positions in reference genome Sscrofa11.1.

### Direct estimation of LD when the four-gamete test fails

Linkage disequilibrium was estimated for consecutive SNPs for which the four-gamete test failed and estimates of ${r}^{2}$ using the standard EM algorithm [5] and the Corners’ Algorithm were compared. Fortran 90 routines were written to perform the EM algorithm and the Corners’ Algorithm and are available at https://github.com/lgomezraya/CORNER

### GWAS for total number of born piglets in Iberian pigs

Association analysis for total number of piglets born was performed using ASReml [22] with the following mixed model:

$$\mathbf{y}= \mathbf{W}\mathbf{b}+\mathbf{X}\mathbf{g}+\mathbf{Z}\mathbf{a}+\mathbf{e},$$

where $\mathbf{y}$ is a vector of records for total number of piglets born; $\mathbf{W}$ is a matrix allocating the fixed effects of parity and farrowing season; $\mathbf{b}$ is a vector of the fixed effects of parity (parity classes 1 to 6, with parity 6 representing 6 or more parities) and farrowing season; $\mathbf{X}$ is a design matrix allocating records to the haplotype effect (modeled as 0, 1, or 2 for homozygous, heterozygous, and alternate homozygous haplotypes, respectively); $\mathbf{g}$ is the fixed effect of the fitted haplotype; $\mathbf{Z}$ is a design matrix allocating records to individuals; $\mathbf{a}$ is a vector of additive values of the sows, assumed to be randomly distributed ${\sim \mathrm{N}(\boldsymbol{0}, \mathbf{G}\upsigma }_{\mathrm{a}}^{2}$), where $\mathbf{G}$ is a genomic relationship constructed using SNP genotype information following VanRaden [23] and ${\upsigma }_{\mathrm{a}}^{2}$ is the additive genetic variance; and $\mathbf{e}$ is a vector of random error.

In single-marker GWAS, each individual is recorded and has 0, 1, or 2 copies of a specific alleles of the evaluated SNP. For two consecutive SNPs, this means that contrasts are made for two haplotypes, one per SNP (Fig. 1). The Corners’ Algorithm allows phasing and testing the effect of the third haplotype against the other two haplotypes, i.e., testing individuals with 0, 1, or 2 copies of that particular haplotype. Thus, we run three GWAS tests for each pair of consecutive SNPs: two tests for the two single SNPs and a third test after phasing the third haplotype with the Corners’ Algorithm (Fig. 1).

The package “qqman” of the R statistical environment [24] was used to create the Manhattan plots [25]. Accounting for multiple testing in GWAS was performed by: (1) controlling the false discovery rate (FDR), defined as the expected proportion of false-positive associations among all associations that were declared significant [26, 27], and (2) reducing the total number of tests performed to partially accommodate LD between consecutive markers as well as the redundancy in testing the three haplotypes after phasing by the Corners’ Algorithm. Note that contrasts are between the performance for one haplotype against the other two, which are correlated because the tests share haplotypes. In total, 20,339 single-marker GWAS tests were performed with 3578 pairs of consecutive SNPs in full LD (two haplotypes segregating). There were 10,380 pairs of consecutive SNPs that failed the four-gamete test (three haplotypes segregating) as detected by the Corners’ Algorithm. Accounting for multiple testing was done with the Benjamini–Hochberg method [28]. This method starts by ordering the *m* tests by ascending *p*-values as estimated in the GWAS procedure. Then, adjusted *p*-values (*P*_*i*_) corresponding to the *i*-th rank of each test are declared significant if ${P}_{i}\le \frac{i}{m}0.05$. That is, genome-wide FDR is set at a significance level of 0.05. This procedure assumes that tests are independent; this assumption is violated since there were SNPs in LD and the tests for haplotypes were not independent. An estimate of the number of effective tests was used instead of the actual number of tests for the calculation of adjusted *p*-values according to the Benjamini–Hochberg method. The effective number of tests (11,630) was the total number of SNPs (20,339) minus half of the SNPs in full LD (3578) and minus two thirds of the tests that failed the four-gamete test (10,380). This approach may still be conservative because the total number of tests included markers that were in partial LD.

## Results

### Exact phasing of haplotypes when the four-gamete test fails: the Corners’ Algorithm

The first step was to identify consecutive markers with maximum LD for the given allele frequencies. The Corners’ Algorithm revealed 10,380 consecutive pairs of SNPs with three haplotypes, which represents about 50% of all pairs of consecutive SNPs.

### Direct estimation of LD when the four-gamete test fails

**Fig. 2:**
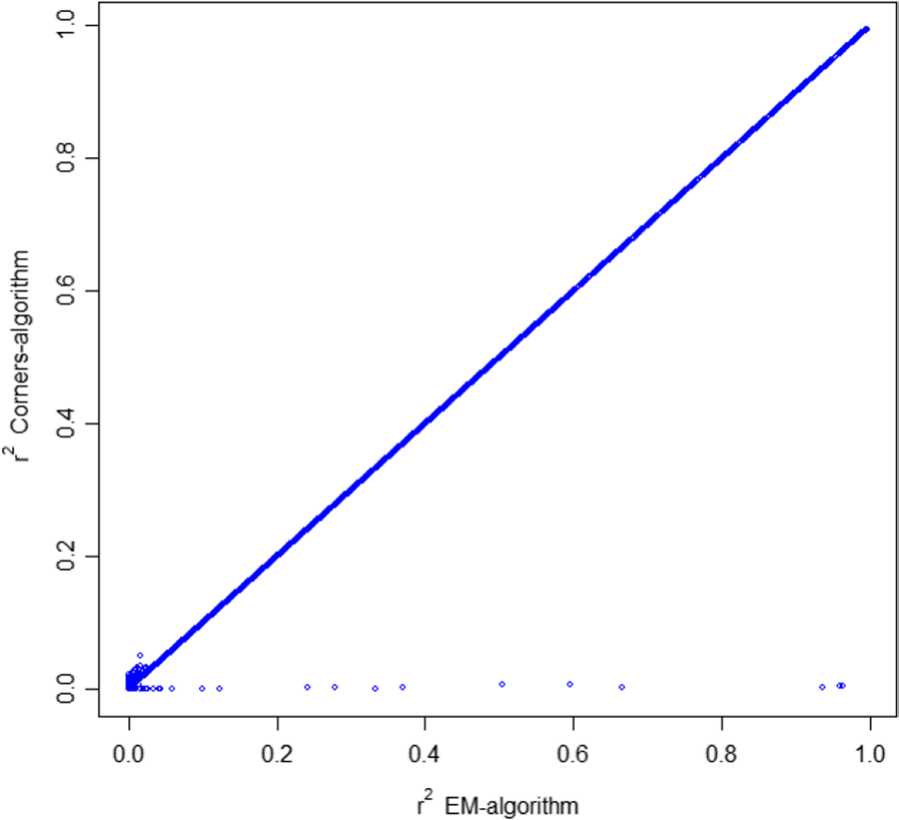
Plot of ${r}^{2}$ estimated with the EM-algorithm versus the Corners’ Algorithm for pairs of SNPs across the genome of the Iberian pig population that failed the four gamete test

Estimation of ${r}^{2}$ was performed using both the EM algorithm and the Corners’ Algorithm for the 10,380 pairs of consecutive SNPs for which the four-gamete test failed. The average of the absolute value of the difference between the two was 0.00082. Figure 2 shows that, regardless of its value, the ${r}^{2}$ estimate was very similar for both methods, except for a few pairs of SNPs with ${r}^{2}$ values close to 0. The largest difference in ${r}^{2}$ estimates was 0.04 or more for 16 pairs of SNPs; in these cases, the genotypic counts showed inconsistencies. For example, SNPs at positions 39,631,490 and 39,638,306 kb on *Sus scrofa* chromosome 2 had just three genotypes *AABB*, *AABb*, and *AaBb* with 382, 17, and 36 individuals, respectively. The estimate of ${r}^{2}$ was 0.002 and 0.665 for the Corners’ Algorithm and the EM algorithm, respectively. The estimates of haplotype frequencies of Corners’ versus EM algorithms were 0.90 versus 0.94 (haplotype *AB*), 0.06 versus 0.02 (haplotype *Ab*), 0.04 versus 0.00 (haplotype *aB*) and 0.00 versus 0.04 (haplotype *ab*). Therefore, the Corner’ Algorithm does not assign any haplotype *ab* to the double heterozygotes whereas the EM algorithm does. Nevertheless, given an equal zygotic mortality for all haplotypes and random mating, one would expect to observe some individuals with genotype *AaBB.* Thus, at least one of the two SNPs in the pair likely had a genotyping error.

**Fig. 3:**
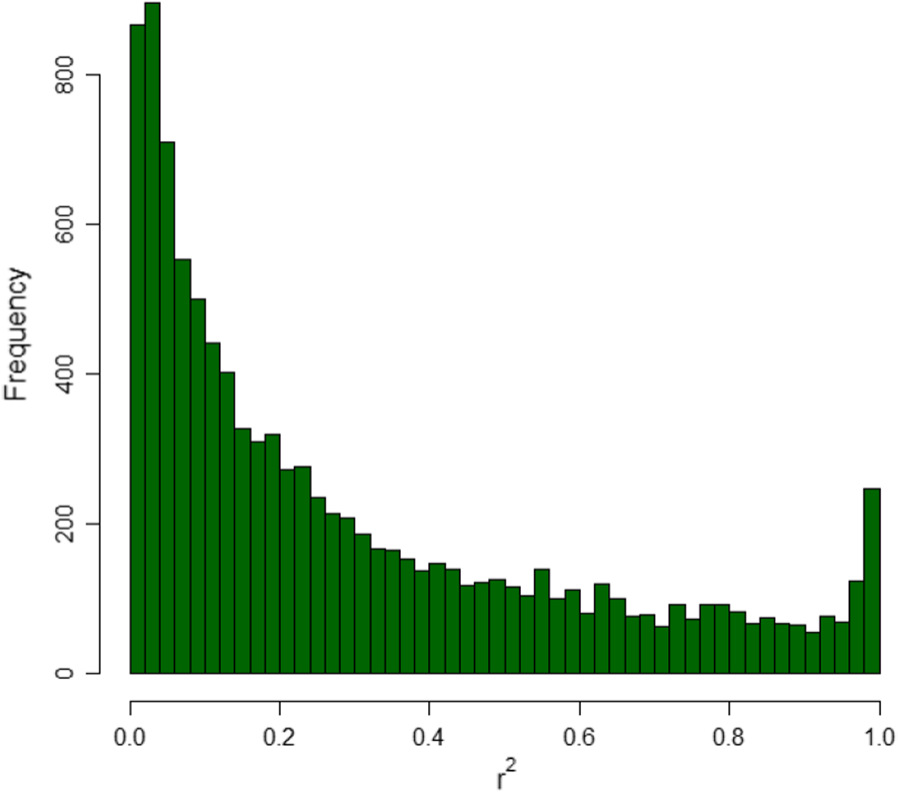
Histogram of ${r}^{2}$ estimated by the Corners’ Algorithm across the genome of the Iberian pig population for pairs of SNPs that failed the four gamete test

Figure 3 depicts a histogram of the distribution of ${r}^{2}$ estimated by the Corner’s Algorithm for consecutive SNPs for which the four-gamete failed. The estimates of ${r}^{2}$ were far from 1 for the majority of these pairs of consecutive SNPs.

### GWAS for total number of born piglets in Iberian pigs

**Fig. 4:**
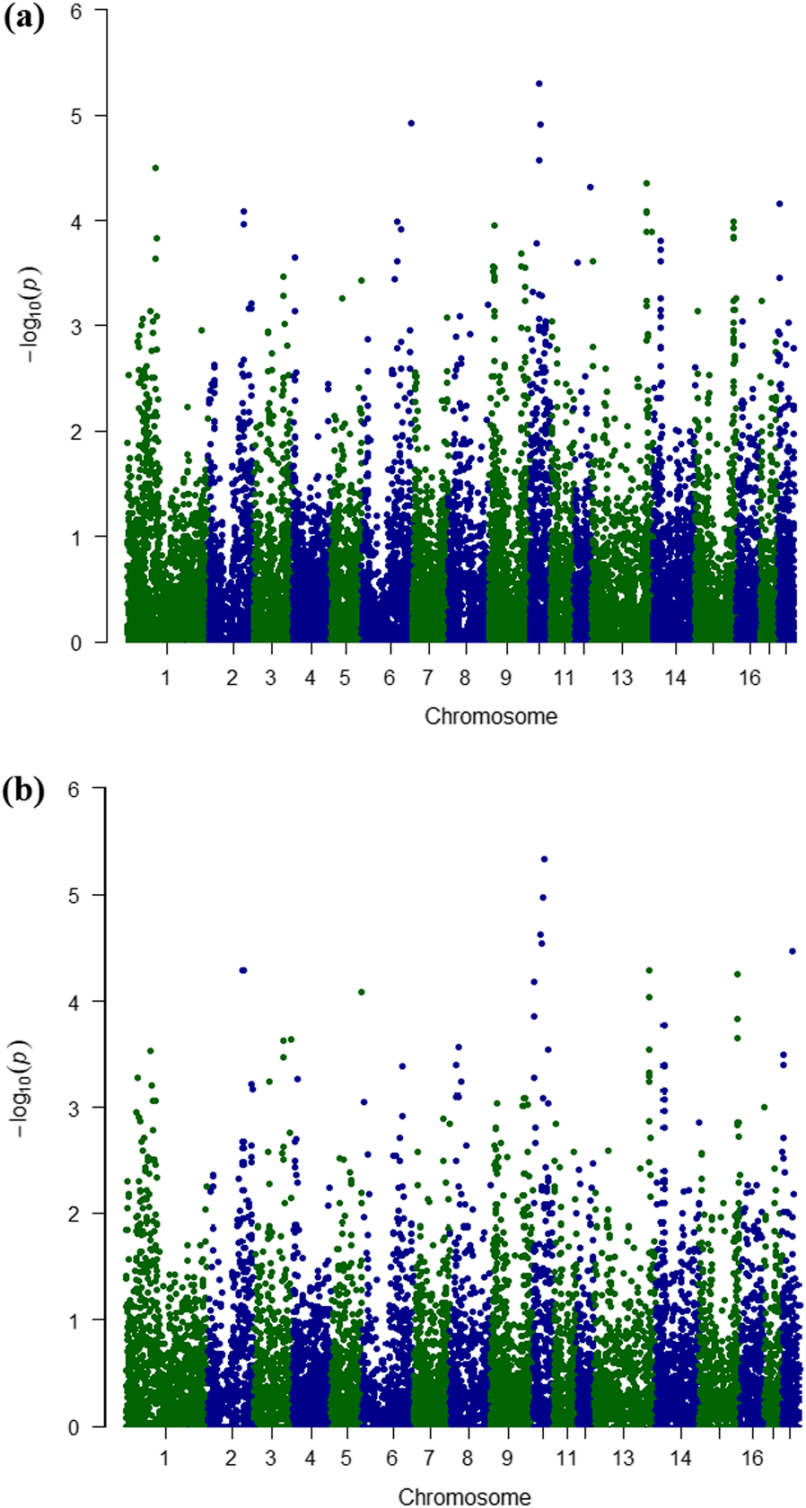
Manhattan plot of genome-wide association analysis (GWAS) for total born piglets in the Iberian pig population: **a** single-marker GWAS and **b** haplotype GWAS of the third haplotype phase-resolved by the Corners’ Algorithm

For pairs of consecutive SNPs for which the four-gamete test failed, we performed a single-marker GWAS for each of the two SNPs, as well as a haplotype-based GWAS for the third haplotype. The Manhattan plot showed some relationships between single marker and haplotype-based GWAS, which is expected because the tests are correlated, as each haplotype is tested against the other two (Fig. 4). Table 3 lists the genome-wide significant results at a significance level of 0.05 (chromosomes 1, 2, 6, 9, 10, 12, 13, 14, 15, and 18). Importantly, some significant haplotypes, on chromosomes 2, 5, 10, 13, 14, 15 and 18 were not detected using the single-marker GWAS. The allele or haplotype effects ranged between 0.40 and 1.35 piglets.

**Table 3:** Results for total number of born piglets using both single-marker and haplotype-based GWAS for pairs of SNPs that failed the four-gamete test

Marker	Chr	bp	p-value	Testing	Effect	SE
MARC0034119	1	96,037,981	5.589E−05	SM	0.4921	0.1137
H3GA0002409	1	99,193,570	1.763E−04	SM	0.4490	0.1142
ASGA0011539	2	118,870,830–118,954,474	7.739E−05	CA	− 0.4590	0.1121
ALGA0103532	2	119,740,603	1.333E−04	SM	− 0.4439	0.1135
ALGA0116789	2	120,068,983	1.032E−04	SM	− 0.4416	0.1110
M1GA0008168	5	101,433,702–101,553,227	9.458E−05	CA	− 0.4321	0.1163
ALGA0110616	6	118,743,309	2.494E−04	SM	− 0.7959	0.2151
MARC0015284	6	119,459,403	1.247E−04	SM	− 0.5651	0.1439
ALGA0115834	6	131,595,818	1.505E−04	SM	0.4338	0.1118
ASGA0084474	6	169,350,462	3.009E−05	SM	1.3460	0.3023
ASGA0103251	9	17,185,238	1.376E−04	SM	− 0.4464	0.1142
ALGA0056412	10	2,642,492–2,657,144	1.634E−04	CA	− 0.6948	0.1806
ALGA0056570	10	5,163,941–5,260,384	8.598E−05	CA	− 0.7179	0.1778
ALGA0057882	10	23,123,837–23,405,449	3.869E−05	CA	− 0.4405	0.1030
ALGA0057882	10	23,123,837	1.892E−04	SM	− 0.4450	0.1169
MARC0093994	10	30,260,928–30,266,906	4.729E−05	CA	− 0.5570	0.1317
ASGA0047467	10	30,266,906–30,272,819	5.159E−05	CA	− 0.5570	0.1317
H3GA0029862	10	31,578,551	4.299E−05	SM	1.0100	0.2372
ASGA0047534	10	32,325,187	8.598E−06	SM	0.6067	0.1311
ASGA0047532	10	32,344,042	1.290E−05	SM	0.6067	0.1311
ASGA0047525	10	32,388,874	1.720E−05	SM	0.6067	0.1311
ASGA0047536	10	32,412,425	2.150E−05	SM	0.6067	0.1311
ASGA0047539	10	32,555,752–32,773,702	2.580E−05	CA	0.4692	0.1051
ALGA0058366	10	33,529,890	3.439E−05	SM	1.1120	0.2505
H3GA0053630	12	12,159,636	2.537E−04	SM	− 0.4482	0.1213
ALGA0067033	12	53,995,960	6.879E−05	SM	0.7064	0.1717
ALGA0067501	13	3,303,385	2.451E−04	SM	0.4340	0.1171
H3GA0037751	13	183,709,477	1.591E−04	SM	0.5516	0.1427
DRGA0013310	13	184,605,970	9.888E−05	SM	0.4426	0.1112
MARC0074099	13	184,896,466	6.449E−05	SM	0.4555	0.1102
MARC0039126	13	185,040,748–185,091,416	1.118E−04	CA	0.4354	0.1101
DRGA0013313	13	185,091,416–185,104,190	1.161E−04	CA	0.4354	0.1101
H3GA0037767	13	185,117,846	1.075E−04	SM	0.4388	0.1104
ASGA0091260	13	186,691,208–186,881,141	7.309E−05	CA	0.4549	0.1109
ALGA0073854	13	201,283,008	1.548E−04	SM	− 0.4747	0.1226
ASGA0062133	14	23,611,137	1.849E−04	SM	− 0.4032	0.1055
DRGA0013731	14	23,697,212–23,832,386	1.935E−04	CA	0.4015	0.1057
ASGA0062259	14	24,043,070	1.978E−04	SM	− 0.4000	0.1060
ASGA0091187	15	128,346,116	1.806E−04	SM	− 0.4183	0.1091
ALGA0087783	15	128,700,470–128,729,028	8.169E−05	CA	− 0.4568	0.1121
MARC0005573	15	129,888,085–130,020,573	1.720E−04	CA	− 0.4458	0.1162
ASGA0091472	15	130,020,573	1.419E−04	SM	− 0.4647	0.1194
SIRI0001312	15	130,037,634	1.462E−04	SM	− 0.4647	0.1194
ASGA0071359	15	131,451,971	1.677E−04	SM	− 0.4215	0.1097
M1GA0020537	15	131,460,883	1.290E−04	SM	− 0.4166	0.1062
ASGA0071383	15	131,638,959	1.204E−04	SM	− 0.4192	0.1068
MARC0019451	18	4,851,570	9.028E−05	SM	− 0.4272	0.1062
ALGA0097920	18	34,356,507–34,392,294	6.019E−05	CA	0.4118	0.1613

Marker: marker name for single marker GWAS or name of first SNP for haplotype-based GWAS; Chr: chromosome; bp: base pair positions; p-value: Benjamini–Hochberg adjusted p-values; Testing: type of testing of haplotype (SM for single-marker GWAS; CA for haplotype-based GWAS using the Corner’s Algorithm for the third haplotype); Effect: effect of haplotype; SE: standard error of the estimate of Effect

### Haplotyping of chromosomal regions that fail the four-gamete test for any combination of SNP pairs

Identification of chromosomal regions that have not undergone recombination can be accomplished by testing the four-gamete test for any possible SNP pair (consecutive and non-consecutive) within that region. If the four-gamete test fails in all cases then the entire region can be unambiguously phased. Table 4 shows the haplotypes for individual 65 for all pairs of SNPs that were resolved with the Corners’ Algorithm for the chromosomal region between bp 309,120 and 1,301,402. The SNPs that matter for phasing are the ones for which this individual is heterozygous. Individual 65 is heterozygous for three SNPs at positions 309,120/1,208,316/1,301,402. After applying the Corner’ Algorithm, the phases for double heterozygotes between 309,120 and 1,208,316 are *AG*/*GA*, and between 309,120 and 1,301,402 are also *AG*/*GA*. Therefore, the haplotypes for these three SNPs are *AGG*/*GAA*. This is confirmed by the phase between 1,208,316/1,301,402 (*GG*/*AA*) in Table 4. Then, alleles at homozygous SNPs are filled in when constructing the full haplotype of the chromosomal region. Thus, the haplotypes of this individual are *AAGCGAAGG* and *GAGCGAAAA*. After haplotyping all individuals, this chromosomal fragment can be considered as a multi-allelic locus site, for which each haplotype represents an allele since no recombination has been observed. Table 5 shows the estimates of the frequencies of the six haplotypes that are segregating in this region. They have a polymorphism information content (PIC) of 0.72 and a heterozygosity of 0.76. A list of all chromosomal regions that failed the four-gamete test is provided in Additional file 1: Table S1 and covered 961.02 of the 2249.32 Mb covered by the SNP array.

**Table 4:** Haplotyping for sow 65 for SNP base pair (bp) positions 309,120, 477,400, 705,066, 712,417, 768,502, 771,992, 887,856, 1,208,216 and 1,301,402 on chromosome 1

bp position	477,400	705,066	712,417	768,502	771,992	887,856	1,208,216	1,301,402
309,120	AA GA	AG GG	AC GC	AG GG	AA GA	AA GA	**AG** **GA**	**AG** **GA**
477,400		AG AG	AC AC	AG AG	AA AA	AA AA	AG AA	AG AA
705,066			GC GC	GG GG	GA GA	GA GG	GG GA	GG GA
712,417				CG CG	CA CA	CA CG	CG CA	CG CA
768,502					GA GA	GA GG	GG GA	GG GA
771,992						AA AG	AG AA	AG AA
887,856							AG AA	AG AA
1,208,216								**GG** **AA**

The underline is the genotype for position 309,120 and is used to illustrate the phasing method. The genotypes beside the underline corresponds to the genotype for the SNP with the position as indicated in the top of the table. Phases of haplotypes for a SNP pair for which the individual is a double heterozygote are in bold and are phased after the Corner’s Algorithm

**Table 5:** Estimates of haplotype frequencies based on the Corners’ Algorithm in a segment on SSC1 with nine SNPs at base pair positions 309,120, 477,400, 705,066, 712,417, 768,502, 771,992, 887,856, 1,208,216 and 1,301,402

Haplotype	Frequency
AAAAAGAAA	0.02183908
AAAAAGGAA	0.07701149
AAGCGAAAA	0.15632184
AAGCGAAGG	0.23218391
AGAAAGAAA	0.14712644
GAGCGAAAA	0.36551724

Polymorphism information content = 0.72, Heterozygosity = 0.76

## Discussion

The present study shows that LD can be estimated directly from haplotype frequencies for pairs of biallelic SNPs for which only three haplotypes are segregating and the four-gamete test fails. The use of iterative methods for haplotype frequency estimation and phasing, such as the EM algorithm of Excoffier and Slatkin [5], is then unnecessary. The EM algorithm is widely used and implemented in standard software such as Haploview [29]. The difference between a direct method to count genotypes and an EM algorithm to estimate LD is that the latter requires starting values of haplotype frequencies that do not necessarily converge to the absolute maximum. Excoffier and Slatkin [5] concluded that, for the EM algorithm, several starting frequencies may be necessary when sample sizes are small. The Corners’ Algorithm infers the phases of double heterozygotes based on the presence of zeroes in corners of the 3 × 3 table of genotype counts at two loci. Note that the probability of missing a haplotype also depends on the sample size and, therefore, both the EM and Corners’ Algorithms may be affected by small sample sizes. For larger sample sizes, our results show that both the EM algorithm and the Corners’ Algorithm gave exactly the same results, except when there are inconsistencies in two-marker genotypes, likely due to genotyping errors. The main advantage of the Corners’ Algorithm is that it facilitates exact phasing of haplotypes. The Corner´s Algorithm is also faster to compute than the EM algorithm since it is based on counting genotypes for pairs of SNPs without the need for iterative mathematical operations.

Failure of the four-gamete test has been used as a method for detecting recombination under the assumption that no back-mutation and/or recurrent mutation exists [9]. We have used a low-density SNP array in this study which may affect the number of tests that failed the four-gamete test. At a higher SNP density, as with data from next-generation sequencing (NGS), recombination events should be identified that would be missed in a low-density array, which would facilitate the construction of fine recombination maps. Another explanation for the abundance of three segregating haplotypes in our data is that the Iberian herd had been closed for many generations with a small population size and high inbreeding [20, 30]. In this context, haplotypes may have been lost by genetic drift.

Methods for phasing haplotypes make use of both LD and familial information that incorporates Mendelian segregation and linkage [31]. In the present study, haplotypes that were fully resolved by the Corners’ Algorithm allowed extension of single-marker GWAS to test all three haplotype effects. Our results illustrate that haplotype variants with an effect on performance that are detected with the Corners’ Algorithm may remain undetected when using single-marker GWAS. This may be one contributing factor to the missing heritability problem [17, 18]. However, identification of the causal mutation remains difficult. In addition, individuals with haplotypes that are bracketed by the same SNP alleles may not share identical DNA sequences within the haplotype fragment, i.e., for SNPs that were not genotyped. Further investigation using DNA sequencing and identifying all polymorphisms is required to reveal all haplotypes that segregate for a given chromosomal fragment and to detect their association with performance.

Genomic selection represents a major progress in methods for genetic improvement of farm animals [32]. One of the most popular applications of genomic selection is genomic best linear unbiased prediction (GBLUP), in which the relationship matrix based on pedigree is replaced by a genomic relationship matrix based on genetic markers [23]. The most popular method to construct genomic relationship matrices uses one SNP at a time when establishing genomic relationships between pairs of individuals. This method ignores haplotype information [23]. The Corners’ Algorithm proposed here could be adapted for genomic selection when three haplotypes are segregating, e.g., by treating each of the three haplotypes as different loci and, by incorporating them in the genomic relationship matrix. This is similar to multi-allelic markers which reduce to biallelic markers by considering one allele versus a pool of the other alleles. However, this may result in incorporating repeated information in the relationship matrix, which requires further research [33].

Haplotyping of chromosomal fragments that fail the four-gamete test for all pairs of SNPs facilitates the use of those haplotypes as alleles in a multi-allelic marker system. This may have a wide range of advantages over existing uses of single SNP information. It can help to identify chromosomal areas with no (or low) recombination when investigating the block structure of the animal genomes [34, 35]. Block determination typically uses a fixed-size sliding window [19] or is based on estimated LD [36]. There have been some contradictory results when using different algorithms for identifying haplotype blocks [37]. Essentially, haplotype blocks are separated from each other by historical recombination events, and, therefore, identifying regions within blocks with no recombination events by the Corners’ Algorithm may be helpful. Once blocks are established, LD between blocks constructed by the Corners’ Algorithm could be estimated using a multi-allelic approach [38, 39].

We also showed that haplotyping a chromosome segment that fails the four-gamete test for all pairs of SNPs (consecutive and non-consecutive) in the segment can be considered as a multi-allelic marker with increased polymorphism information content (PIC) and heterozygosity, which may be useful in a variety of scenarios such as paternity analysis, traceability, and DNA forensics. Ultimately, it can be used for finding local molecular relationships within and between populations that share non-recombinant haplotypes, as identified by the Corners’ Algorithm.

## Conclusions

The Corners’ Algorithm allows to fully phase-resolve the haplotypes for all animals for SNPs for which the four-gamete test fails. Direct equations to estimate LD for such SNP pairs are provided that can replace iterative algorithms such as the EM algorithm. The resulting three haplotypes that are fully phase-resolved for all individuals can be implemented in GWAS, extending the testing of single-marker GWAS to haplotype-based GWAS. Haplotyping of chromosomal fragments that fail the four-gamete test for all SNP pairs can be used as a multi-allelic marker to increase PIC, elucidate haplotype blocks and reveal local historic relationships within and between populations.

### Supplementary Information

**Additional file 1: Table S1.** Non-recombination chromosomal regions in Iberian pigs detected by the Corners’ Algorithm.

## Data Availability

The genotyping data used are available in Zenodo (https://doi.org/10.5281/zenodo.6636369).

## Direct LD estimation using the Corner’s Algorithm

### Missing haplotype *Ab*

Estimation of ${r}^{2}$ when haplotype *Ab* is missing can be deducted based on Table 2 and by computing haplotype frequencies by using only genotype counts corresponding to unambiguous haplotypes. Double heterozygotes must have phase *AB/ab*. Thus:

$${f}_{AB}=\frac{{n}_{AaBb}+2{n}_{AABB}+{n}_{AABb}+{n}_{AaBB}}{2N},$$

$${f}_{Ab}=0,$$

$${f}_{aB}=\frac{2{n}_{aaBB}+{n}_{AaBB}{+n}_{aaBb}}{2N},$$

$${f}_{ab}=\frac{2{n}_{aabb}+{n}_{aaBb}{+n}_{Aabb}{+n}_{AaBb}}{2N},$$

$${f}_{A}=\frac{2{n}_{AA}+{n}_{Aa}}{2N},$$

$${f}_{a}=\frac{2{n}_{aa}+{n}_{Aa}}{2N},$$

$${f}_{B}=\frac{2{n}_{BB}+{n}_{Bb}}{2N},$$

$${f}_{b}=\frac{2{n}_{bb}+{n}_{Bb}}{2N},$$

with $${n}_{AA}={n}_{AABB}+{n}_{AABb}+ {n}_{AAbb}$$;$${n}_{Aa}={n}_{AaBB}+{n}_{AaBb}+ {n}_{Aabb}$$; $${n}_{aa}={n}_{aaBB}+{n}_{aaBb}+ {n}_{aabb}$$; $${n}_{BB}={n}_{AABB}+{n}_{AaBB}+ {n}_{aaBB}$$;$${n}_{Bb}={n}_{AABb}+{n}_{AaBb}+ {n}_{aaBb}$$; $${n}_{bb}={n}_{AAbb}+{n}_{Aabb}+ {n}_{aabb}$$; $$N= {n}_{AA}+{n}_{Aa}+{n}_{aa}={n}_{BB}+{n}_{Bb}+{n}_{bb}$$.

Substituting in Eq. (1) yields:

$${r}^{2}={\left[\frac{({n}_{AaBb}+2{n}_{AABB}+{n}_{AABb}+{n}_{AaBB})(2{n}_{aabb}+{n}_{aaBb}{+n}_{Aabb}{+n}_{AaBb})}{\sqrt{(2{n}_{AA}+{n}_{Aa})(2{n}_{aa}+{n}_{Aa})(2{n}_{BB}+{n}_{Bb})(2{n}_{bb}+{n}_{Bb})}}\right]}^{2}.$$

### Missing haplotype *aB*

Estimation of ${r}^{2}$ when haplotype *aB* is missing can be deducted after using Table 2, and by computing haplotype frequencies by using only genotype counts corresponding to unambiguous haplotypes. Double heterozygotes must have phase *AB/ab*. Thus:

$${f}_{AB}=\frac{2{n}_{AABB}+{n}_{AABb}+{n}_{AaBB}+{n}_{AaBb}}{2N},$$

$${f}_{Ab}=\frac{2{n}_{AAbb}+{n}_{AABb}{+n}_{Aabb}}{2N},$$

$${f}_{aB}=0,$$

$${f}_{ab}=\frac{2{n}_{aabb}+{n}_{aaBb}{+n}_{Aabb}+{n}_{AaBb}}{2N},$$

$${f}_{A}=\frac{2{n}_{AA}+{n}_{Aa}}{2N},$$

$${f}_{a}=\frac{2{n}_{aa}+{n}_{Aa}}{2N},$$

$${f}_{B}=\frac{2{n}_{BB}+{n}_{Bb}}{2N},$$

$${f}_{b}=\frac{2{n}_{bb}+{n}_{Bb}}{2N},$$

which after substituting these frequencies in Eq. (1) yields:

$${r}^{2}={\left[\frac{(2{n}_{AABB}+{n}_{AABb}+{n}_{AaBB}+{n}_{AaBb})(2{n}_{aabb}+{n}_{aaBb}{+n}_{Aabb}+{n}_{AaBb})}{\sqrt{(2{n}_{AA}+{n}_{Aa})(2{n}_{aa}+{n}_{Aa})(2{n}_{BB}+{n}_{Bb})(2{n}_{bb}+{n}_{Bb})}}\right]}^{2}.$$

### Missing haplotype *ab*

Estimation of ${r}^{2}$ when haplotype *ab* is missing can be deducted after using Table 2, by computing haplotype frequencies by using only genotype counts corresponding to unambiguous haplotypes. Double heterozygotes must have phase *Ab/aB*. Thus:

$${f}_{AB}=\frac{2{n}_{AABB}+{n}_{AABb}+ {n}_{AaBB}}{2N},$$

$${f}_{Ab}=\frac{2{n}_{AAbb}+{n}_{AABb}+ {n}_{Aabb}+{n}_{AaBb}}{2N},$$

$${f}_{aB}=\frac{2{n}_{aaBB}+{n}_{AaBB}{+n}_{AaBb}+{n}_{aaBb}}{2N},$$

$${f}_{ab}=0,$$

$${f}_{A}=\frac{2{n}_{AA}+{n}_{Aa}}{2N},$$

$${f}_{a}=\frac{2{n}_{aa}+{n}_{Aa}}{2N},$$

$${f}_{B}=\frac{2{n}_{BB}+{n}_{Bb}}{2N},$$

$${f}_{b}=\frac{2{n}_{bb}+{n}_{Bb}}{2N},$$

which after substituting these frequencies in Eq. (1) yields:

$${r}^{2}={\left[-\frac{(2{n}_{AAbb}+{n}_{AABb}+ {n}_{Aabb}+{n}_{AaBb})(2{n}_{aaBB}+{n}_{AaBB}{+n}_{AaBb}+{n}_{aaBb})}{\sqrt{(2{n}_{AA}+{n}_{Aa})(2{n}_{aa}+{n}_{Aa})(2{n}_{BB}+{n}_{Bb})(2{n}_{bb}+{n}_{Bb})}}\right]}^{2}.$$
